# The Current State of Palliative Care Research for Adolescents and Young Adults With Cancer: A Systematic Review and Meta‐Thematic Analysis of Empirical Literature

**DOI:** 10.1002/pon.70228

**Published:** 2025-07-14

**Authors:** Anao Zhang, Meiyan Chen, Rachel Brandon, Jennifer Currin‐McCulloch, Xingzhi Liu, Brennan Cheladyn, Betty Ferrell, Barbara Jones, Bradley Zebrack, Adam S. DuVall

**Affiliations:** ^1^ University of Michigan School of Social Work Michigan Medicine AYA Oncology Program Ann Arbor Michigan USA; ^2^ The University of Texas at Austin School of Social Work Austin Texas USA; ^3^ University of Michigan School of Social Work and Department of Psychology Ann Arbor Michigan USA; ^4^ Colorado State University School of Social Work Fort Collins Colorado USA; ^5^ Michigan Medicine AYA Oncology Program Ann Arbor Michigan USA; ^6^ City of Hope Medical Center Duarte California USA; ^7^ Boston University School of Social Work Boston Massachusetts USA; ^8^ Department of Medicine Assistant Professor of Medicine and Assistant Professor of Pediatrics University of Chicago Chicago Illinois USA

**Keywords:** adolescent and young adult cancer, meta‐thematic analysis, palliative care, systematic review

## Abstract

**Background:**

Palliative care is critical to the wellness of cancer patients and survivors, but adolescents and young adults (AYAs) with cancer are confronted with the divide between pediatric and adult palliative care as well as pediatric and adult/medical oncology. Consequently, attention to palliative care for AYAs with cancer has just started, and our understanding of palliative care for AYA oncology remains limited.

**Aim:**

This study aims to update our understanding of this salient topic through rigorous research synthesis.

**Design:**

A systematic review of empirical literature was adopted, and data was innovatively analyzed using meta‐thematic analysis. The review was pre‐registered on OSF (https://osf.io/zy9cs). Studies were searched across ten electronic databases from inception to August 1, 2024 (i.e., the search date). Eligibility criteria include: (1) study the topic of palliative care; (2) focus on AYA cancer; and (3) report empirical data. Meta‐thematic analysis and reflexive thematic analysis informed data analysis methods.

**Results:**

Fifty‐six primary studies from 2004 to 2024, inclusive of 478 data points, were included in the meta‐thematic analysis. Five broad themes were identified: (1) the overall state of AYA palliative care; (2) key characteristics of palliative care for AYAs with cancer; (3) AYA palliative care communication, decision‐making, and AYA involvement; (4) prevalent symptoms, care preferences, and priorities in palliative care among AYAs with cancer; and (5) effective interventions.

**Conclusions:**

Key insights regarding AYA cancer palliative care have been revealed. Notable areas of improvement regarding the delivery of palliative care to AYAs and future research directions have been identified.

## 1 Introduction

Adolescents and young adults (AYAs) with cancer are an age‐defined group, referring to those who received their initial cancer diagnosis between 15 and 39 years old [1]. In the United States, more than 90,000 AYAs are diagnosed with cancer annually, and a recent study estimated over 2.1 million survivors of AYA cancer are residing in the U.S. [2, 3]. In addition to the common challenges confronting individuals with cancer of all ages, AYAs with cancer are uniquely vulnerable to a host of biopsychosocial challenges due to the disruptions to numerous developmental milestones both during and after active cancer treatment [4, 5]. A robust body of literature has revealed that challenges such as onco‐fertility, sexuality, financial toxicity, academic and occupational disruptions, and the cancer care division between pediatric and adult medical oncology disproportionately impact the wellness of AYAs with cancer [4, 5, 6].

Notably, AYAs with cancer often report worse outcomes than their pediatric or adult counterparts. For example, studies have found that survivors of AYA cancers are at significantly greater risk of developing subsequent cancers and dying from recurrence or progression of their primary cancer than childhood cancer survivors [7]. Similarly, when compared with older patients with cancer, AYAs have significantly greater risks of long‐term and late effects, resulting in worse mental health and compromised quality of life [7, 8, 9, 10]. Therefore, it is critical that oncological providers deliver high‐quality care to the AYA cancer population, including high‐quality palliative care.

Palliative care, according to the World Health Organization (WHO), “is an approach that improves the quality of life of patients and their families facing the problems associated with life‐threatening illness, through the prevention and relief of suffering by means of early identification and impeccable assessment and treatment of pain and other problems, physical, psychosocial and spiritual.” [11] The AYA cancer population faces a divide between pediatric and adult palliative care, similar to the division between pediatric and adult medical oncology [12, 13]. As a result, an AYA cancer patient's access to palliative care and the type of palliative care services available to them are largely determined by whether they are treated in a pediatric setting or not [14, 15]. In a recent study that evaluated the palliative care needs in AYAs with chronic health conditions [16], researchers found that the transition to adult palliative care is difficult and poorly facilitated for young patients with complex illnesses, including cancer. Similarly, a qualitative study interviewing palliative care clinicians revealed unique challenges of delivering palliative care to AYAs with advanced cancer, including the uncertainty regarding how to involve family members due to the transitional age (from minor to adult) of the AYA population [17]. Taken together, an overwhelming body of literature has highlighted the clinical significance of palliative care to AYAs with cancer, yet research on palliative care for the AYA cancer population remains limited.

Conceptual papers on palliative care in AYAs with cancer were initially published over two decades ago, with several reviews discussing palliative care in the context of AYA oncology [18, 19, 20, 21]. Rosenberg and Wolfe [19], for example, reviewed core concepts in AYA cancer and palliative care as well as the scope of palliative care in AYA oncology. Pritchard and colleagues [20] shared the Canadian perspectives regarding when, how, and where to introduce palliative care for AYAs' psychosocial needs. Several additional reviews have recently updated our understanding of palliative care for AYAs with cancer [14, 22, 23, 24, 25]. Consistently, these updated reviews documented the clinical and research progress in palliative care for AYAs with cancer while underscoring that research evidence for the AYA oncology population is still in the early stage. Therefore, it is critical to evaluate the current state of research focusing on palliative care in AYA oncology.

In addition to conceptual/narrative reviews, our literature search identified one existing scoping review by Drake and colleagues [24], with a notable focus on the delivery of palliative and end‐of‐life care to AYAs with cancer. This scoping review “identified knowledge gaps and [described and] discussed the key characteristics and types of evidence in [palliative care for AYAs with cancer]” (p. 611) [24]. This current paper meaningfully complements and extends the work of the referenced scoping review by (1) systematically reviewing all empirical research evidence (not just that pertaining to delivery) of palliative care to AYAs with cancer, and (2) analyzing the existing evidence using a meta‐thematic analytical framework. The two primary guiding questions are: “*What is the current state of empirical research on palliative care for AYAs with cancer?*” and “*Is there any empirically derived agreement or disagreement on topics regarding palliative care for AYAs with cancer?*”

## 2 Methods

### 2.1 Design

A systematic review of studies reporting empirical data on palliative care in the AYA cancer population was undertaken in August 2024 following the guidance for conducting systematic scoping reviews [26]. The study protocol was prospectively registered with the Open Science Framework (https://doi.org/10.17605/OSF.IO/ZY9CS). A team of five researchers followed a protocol‐specific screening process in study selection. Using a pre‐designed coding sheet, data was extracted, analyzed, and synthesized using an (inductive) meta‐thematic approach [27, 28]. Given the palliative care focus of the study, we chose Braun and Clarke's recent methodological insights for performing reflexive thematic analysis in palliative medicine and their reflective thematic analysis reporting guidelines to frame our interactions with the data and thematic synthesis [29]. We used the Preferred Reporting Items for Systematic Reviews and Meta‐analysis (PRISMA) [30] to guide the reporting of results.

### 2.2 Research Questions

(1) What topic areas on palliative care in the AYA cancer population have been evaluated by empirical studies? and (2) If any topic area emerges, what is the latest understanding of that topic as reported by the empirical literature?

### 2.3 Eligibility Criteria

To be eligible for inclusion, a primary study was required to: (1) study the topic of palliative care, which is determined by the use of relevant keywords in primary studies, that is, palliative care (or palliation), end‐of‐life care, hospice, or advance care plan; (2) focus on the AYA cancer population (i.e., participants 15–39 years old) per the National Cancer Institute standard [31]; and (3) report empirical data from a broad range of study designs, including but not limited to randomized controlled trials, electronic health record reviews, descriptive studies of patient‐reported data or qualitative studies, among others. Our use of the 15–39 age range best represents the AYA age definition in the United States, whereas there exists much variation globally. We chose to adopt the 15–39 age range because it is the broadest and most inclusive range, and it befits the purpose of this study. Studies were excluded if they (1) focused on supportive cancer care outside the context of palliative care (e.g., psychosocial support *without* referencing concurrent palliative care); (2) targeted pediatric or adult cancer patients/survivors who are not AYAs; (3) only focused on providers without including patients, survivors, or caregivers in the study; and (4) were non‐empirical, that is, theoretical or conceptual reports on palliative care in AYAs without empirical data. We decided to exclude studies only focused on providers without patients, survivors, or caregivers because, first, we only identified two provider‐focused studies and, second, we wanted to maintain the study's focus on patient/survivor‐ and family‐centered outcomes and evidence.

### 2.4 Search Strategy and Information Sources

In consultation with an information scientist at the Library of the University of Michigan, we used a pre‐determined set of keywords to search across eight electronic databases (via EBSCO host), Medline, and the Cochrane Library. Except for using the MeSH terms for Medline search, we used a combination of free texts (as keywords) and Boolean Logic operators to conduct title/abstract searches in EBSCO host and Cochrane Library. The keywords used to conduct the search were the same for EBSCO host across eight databases and the Cochrane Library (see Supporting Information S1). We searched for all studies from inception to August 1, 2024 (i.e., the date of the search). The reference lists of all included studies and other relevant reviews were screened to identify potentially relevant studies. Gray literature was searched using professional websites and the ProQuest dissertation databases for unpublished dissertation manuscripts (see Supporting Information S1). A detailed protocol is provided in Supporting Informtion S1.

### 2.5 Screening Procedures

All identified records were imported into Covidence—a Cochrane‐recommended systematic review platform. After duplicates were removed by the system, the remaining studies went through title/abstract screening and then full‐text screening against the pre‐set inclusion criteria. At every stage, each article was screened/reviewed by two independent reviewers (AZ, MC) with a 98% and 96% agreement between the two screeners/reviewers for title/abstract and full‐text screening, respectively. All disagreements were resolved through discussions. Two independent researchers (MC, RB) independently extracted information from each included study, and all extracted information was verified again by the lead author (AZ) for accuracy. All three screeners are either doctoral students or doctoral degree holders with a substantive interest in supportive care for AYAs with cancer, including palliative care.

### 2.6 Quality Rating

Given the heterogeneous design of included studies, quality rating used tools recommended by the National Heart, Lung, and Blood Institute (https://www.nhlbi.nih.gov/health‐topics/study‐quality‐assessment‐tools), including a collection of tools for quality assessment across various study designs, for example, controlled intervention studies, observational cohort and cross‐sectional studies, and case series studies, among others. Our team matched the specific design of each article with its corresponding quality assessment tools.

### 2.7 Data Synthesis

In addition to descriptive statistics of bibliographic information, meta‐thematic analysis [27, 28] informed by reflexive thematic analysis guidelines (in palliative medicine) [29] was used to analyze empirical findings across included studies. Our primary analytical team, comprising an experienced researcher in psychosocial and palliative AYA oncology, a research assistant from the AYA oncology program with a science background, and a survivor of AYA cancer, employed a reflective approach to thematic analysis. We convened to thoroughly discuss the project's purpose and context. A key part of our pre‐analysis process was reflecting on how our positionalities might impact our interpretations and categorizations of the empirical findings. We adhered to the Reflexive Thematic Analysis Reporting Guidelines and, given the project's purpose, emphasized a post‐positivist perspective. The lead author approached the data with an open mindset, allowing the findings to self‐organize/group inductively without imposing a preconceived theoretical framework. The other team members adopted a similar approach, with the AYA cancer survivor reflecting on how his embodied researcher identity may shape/form his interpretation and understanding of the study findings. Data analysis proceeded in four primary steps.

First, we extracted empirical findings verbatim from individual studies, and each individual finding was treated as a data point. We utilized Miro, an online collaboration software program, to arrange and visualize data according to topics within and then between studies. The software also enabled us to create a final clustering of topics into themes. Second, with each data point, we conducted a level‐two coding of themes by extracting the broader topics presented within the data point. For example, one of the findings [32] (verbatim) was that “more than three‐quarters of AYA patients received at least one form of intensive end‐of‐life care (78%), including chemotherapy within 14 days of death …” For this data point, its topic was coded “*many/most AYAs receive aggressive disease focused end‐of‐life care, chemotherapy.*” Third, individual data points were then grouped inductively based on similarities between the shared meanings developed through level‐two coding. Finally, we labeled similar data points using themes/subthemes to summarize overall categories of findings across empirical studies. Two coders (AZ, XL) independently conducted each step of coding, and disagreements were resolved through discussion. Importantly, an undergraduate pre‐health major research assistant who is a survivor of AYA cancer (BC) reviewed all the grouping and labeling of themes as a critical step in engaging member reflection and insight. The project team used the Miro platform (www.miro.com/app)—an online collaborative whiteboard software—to facilitate all stages of data synthesis.

## 3 Results

### 3.1 Search Results

**FIGURE 1:**
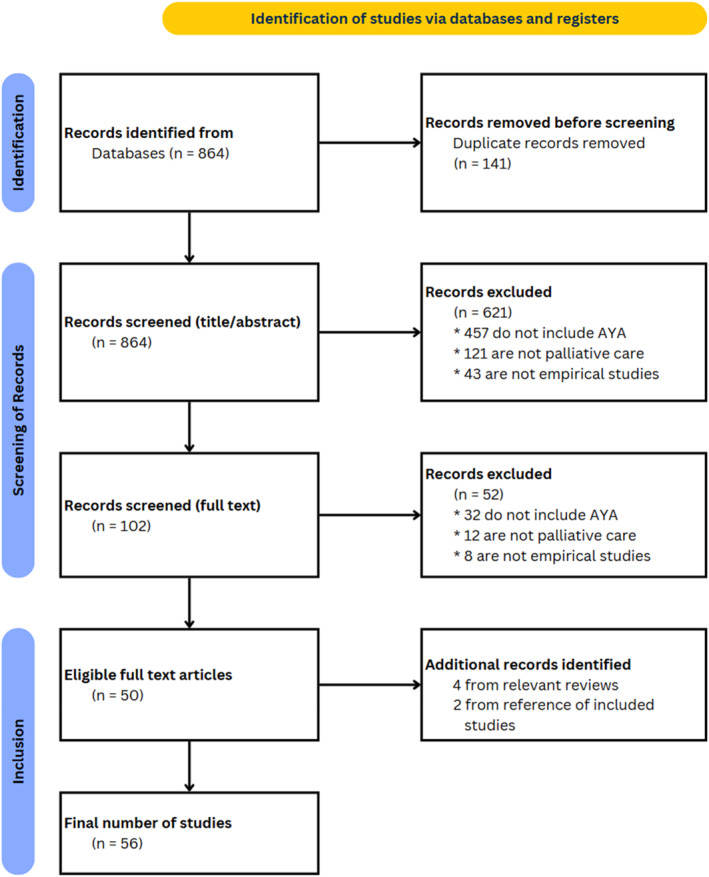
PRISMA chart.

The search strategy resulted in an initial 864 entries of references/studies being imported into Covidence. With Covidence identifying 141 duplications, 723 unique references/studies were included for title and abstract screening. Three screeners (AZ, MC, RB) independently screened these articles, with each article's eligibility being reviewed by at least two screeners, leaving 102 articles for full‐text screening. The full‐text review excluded 52 articles for various reasons, resulting in 50 unique articles, that is, primary studies, for inclusion from electronic databases. In addition, our manual search of existing reviews identified six additional articles for inclusion, resulting in a final analytical sample of 56 primary studies (see PRISMA in Figure 1).

### 3.2 Key Characteristics and Quality Rating of Included Studies

**FIGURE 2:**
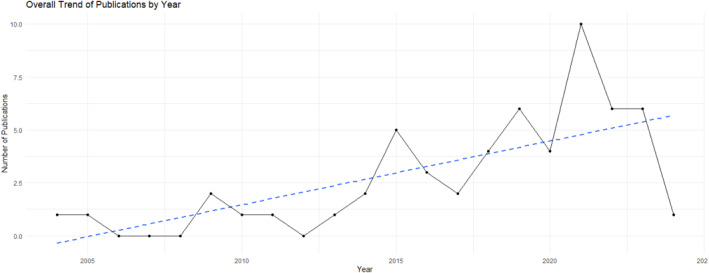
Overall study publication trend.

Of the 56 studies, most primary studies were chart reviews (*n* = 26, 46.4%) and survey studies (*n* = 10, 17.9%), followed by nine using qualitative interviews (16.1%), eight clinical trials (14.3%), one mixed method study, one quality improvement project, and one text analysis study. Studies were published from 2004 to 2024, with an overall increasing trend of study numbers (Figure 2). Most primary studies were published in the United States (*n* = 32), followed by Canada (*n* = 6), Australia (*n* = 5), Japan (*n* = 3), France (*n* = 3), United Kingdom (*n* = 2), Belgium (*n* = 1), Mexico (*n* = 1), South Korea (*n* = 1), Australia and Brazil (*n* = 1), and one transnational (United Kingdom, Germany, and Australia). Most studies (*n* = 36, 64.3%) focused only on AYAs with cancer, eight studies (14.3%) focused on AYA patients/survivors, their parents/caregivers, and providers, six studies (10.7%) focused on AYAs and their parents, leaving six (10.7%) focused on parents/caregivers only.

Out of the 52 studies that reported the AYA age range, 24 used an upper age limit of 39 years old, and the age ranges were heterogeneous across studies (presented in Table S1). Due to the different types of studies included, study sample sizes ranged from five to 30,573, with few studies reporting the participants' gender composition. Thirty‐one studies reported percentage of White AYAs with cancer, with an average of 63.4% of all AYAs being non‐Hispanic White. A frequency analysis of the keywords revealed that most studies included the keywords “end‐of‐life” (*n* = 43) and “palliative care” (*n* = 40), followed by “pain management” (*n* = 17), “hospice” (*n* = 16), “advance care plan” (*n* = 16), and “advanced cancer” (*n* = 9). Of the 56 included studies, only 10 explicitly defined the term palliative care, and the definitions used are presented in Table S1. Most studies reported low risks of bias, that is, high quality, followed by 10 studies reporting moderate risks of bias and two studies reporting high risks of bias.

### 3.3 Meta‐Thematic Findings

A total of 56 primary studies, including 478 data points, were included in the meta‐thematic analysis. Our analysis derived four broader themes, including (1) Navigating the Quality and Accessibility Barriers of Palliative Care in AYAs with Cancer; (2) Too Late and Too Much (Intensive Measures of) Care Delivered; (3) [The Importance of] AYAs' Having a Voice in Palliative Care; and (4) AYAs Choose Comfort and Peace for Palliative Care, Especially End‐of‐Life Care.

#### 3.3.1 Theme One: Navigating the Quality and Accessibility Barriers of Palliative Care in AYAs With Cancer

##### 3.3.1.1 Subtheme 1: It is Challenging to Access Palliative Care, Including End‐of‐Life Care

Overall, 11 primary studies (19 empirical findings) suggested that AYAs with cancer and their families have poor access to palliative care, with emerging evidence suggesting health disparities being notable concerns [12, 33, 34, 35, 36, 37, 38, 39, 40, 41, 42]. For example, Jacobs et al. [35] reported that “*the majority (71%) of the adolescents had never heard about or completed an advanced directive such as the Five Wishes document,*” and “*53% stated that they had never talked with anyone about their EOL preferences.*” Similarly, Montel et al. [33] revealed that “*three out of 21 (14%) families had access to palliative care …*” and *“[less than half of the] families had access to home hospitalization.*” Importantly, four studies (and seven findings) identified health disparities in AYA cancer survivors' access to palliative care. Roeland et al. [38] revealed that “… AYA patients living in low‐income zip codes were found to be less likely to enroll in hospice (*p* ≤ 0.01) or receive EOL cancer treatment (*p* ≤ 0.03).” However, this emerging subtheme on health disparity remains preliminary, given the small number of empirical studies and findings.

##### 3.3.1.2 Subtheme 2: Quality Indicators in Palliative Care Are Lacking

In addition to poor access, nine primary studies and 15 empirical findings suggested low quality of AYA palliative care [33, 34, 39, 40, 42, 43, 44, 45, 46]. Important indicators used in primary studies to evaluate the quality of palliative care included, but were not limited to, a record of preferred place of death, a record of do‐not‐resuscitate orders, general documentation (e.g., any documentation at all, ambiguity, coding), and stakeholder perceived achievement of “a good death.” Studies, for example, reported that “*for most (n = 54, 71%) [AYA cancer] patients, preferred place of death was not discussed, not documented, or not expressed by patients*” [43] and that “*[a lot more,* that is*,] 92.6% AYA patients did not have a documented DNR [versus] 78% of the adult group…*” [45]. Similarly, another study evaluated palliative care quality perceived by bereaved Japanese family members and documented that “*overall, [bereaved family members reported] young cancer patients did not achieve a good death*” and “*lower achievement of a good death was significantly correlated with younger age of the bereaved families.*” [44].

#### 3.3.2 Theme Two: Too Late and Too Much (Intensive Measures of) Care Delivered

##### 3.3.2.1 Subtheme 1: Delays Lead to Missed Opportunities for (Timely) Palliative Care Intervention

Fourteen primary studies, including 21 empirical findings, revealed that the timing of palliative care is often delayed for AYAs with cancer, with emerging evidence suggesting poor patient‐parent congruence on the best timing of introduction [32, 34, 35, 36, 37, 38, 42, 43, 47, 48, 49, 50, 51, 52]. Numerous studies have shown, for example, “*[palliative care] consults occurred in a median of 401 days after diagnosis of malignancy*” [42], and “*96% of the patients had documented care preferences [only] in the last month of life*” [32]. Several studies considered factors associated with palliative consultation and timing, revealing that having an Intensive Care Unit (ICU) stay and having an integrated palliative care service team were significantly associated with an (early) palliative consultation [36, 52].

##### 3.3.2.2 Subtheme 2: Escalated/Intensive Care Measures/Procedures in AYA Palliative Care, Resulting in Compromised Care Quality

A total of 28 primary studies, including 151 empirical findings, extensively documented the high intensity of AYA palliative care, which was operationalized in primary studies as (1) aggressive or intensive life‐sustaining measures at end of life or (2) emergency department visit or hospitalization or ICU admission in last month of life [32, 36, 37, 39, 40, 41, 42, 45, 48, 49, 50, 51, 53, 54, 55, 56, 57, 58, 59, 60, 61, 62, 63, 64, 65, 66, 67, 68]. For example, Johnston et al. [41] found that “*more than half of the patients (59%) received at least one inpatient high‐intensity end‐of‐life intervention, and 30% received two or more.*” Similarly, other studies found that “*three‐quarters of all AYA Medicaid decedents received at least 1 intensive measure at the end‐of‐life*” and “*the most common end‐of‐life interventions [for children, adolescents, and young adults with hematologic malignancies] include mechanical ventilation, CPR, and intravenous chemotherapy.*” [50] Importantly, 10 primary studies (and 16 empirical findings) identified that most AYAs are more likely to die in a hospital or with high bed utilization, reflecting that AYAs are more susceptible to aggressive/intensive measures at end of life [39, 42, 49, 55, 56, 60, 61, 64, 65, 68]. For example, Snaman et al. [69] found that *“46% of [AYAs with cancer] died in the intensive care unit.*”

Two factors have been consistently linked with increased intensive procedures used in palliative care, that is, a hematological diagnosis and social determinants of health. Twenty‐three empirical findings revealed that hematologic malignancies are associated with aggressive/intensive measures and procedures in palliative care. For example, “*patients with hematologic malignancies, such as acute myelogenous leukemia, were more likely to die in the hospital”* [55] versus solid tumor patients. In addition, eight empirical findings identified social determinants of health associated with high‐intensity palliative care, that is, rurality, insurance status, low income, and racial and ethnic minority. For example, Coltin et al. [62] found that “*AYAs with cancer living in rural areas at diagnosis were more likely to die in the ICU,*” and Revon‐Riviere et al. [43] reported that “*high‐intensity end‐of‐life care was predicted by [an AYA cancer patient's] social disadvantage”* as measured by the deprivation index.

An emerging, though preliminary, overall finding within this subtheme is that AYAs strongly prefer to die at home; however, many die in the hospital. Studies have found that “*94% of teens [with cancer] prefer being able to stay in [their]own home [when dying]*,” [35] or “*74.1% AYAs [from Australia] preferred a home death, … as compared to only 45.9% of adults*” [45]; and studies also reported “*[close to half] 44% [of AYA cancer patients] died in hospital…*” [40] and “*[AYA] patients residing within 5 miles of a specialty center were more likely to die in a hospital [versus those who lived > 20 miles from a hospital]*”.

##### 3.3.2.3 Subtheme 3: Complex Structure of Palliative Care Referral Patterns

Fourteen primary studies, including 40 empirical findings, reported clinical and disease‐related factors that impact the pattern of palliative care referrals [34, 37, 38, 40, 41, 42, 43, 50, 51, 56, 58, 61, 70, 71]. Specifically, cancer diagnosis and treatment setting were identified as two key factors impacting palliative care referral. Interestingly, while some studies found “*hematological malignancies were the most prevalent cancer types in the AYA group*” [71] and “*[both] leukemia and non‐Hodgkin lymphoma are the [top] five‐most common cancer types for AYAs with palliative care referrals*” [34], more studies revealed that “*patients with hematologic malignancies had lower odds of a hospice referral than those with solid tumors*” [58], or “*patients with hematologic malignancies had lower odds of hospice referrals*” [38].

Inpatient settings are associated with increased palliative care referrals, with studies reporting “*the presence of a documented do‐not‐resuscitate order was significantly associated with inpatient death*” [61] and “*of patients with palliative care consults, there were on average more inpatient versus outpatient encounters*” [42]. Other identified factors included being non‐Hispanic White, having public insurance, and being female, which are all associated with increased palliative care referral for AYAs with cancer, though they remain preliminary, and we did not identify them as a theme.

#### 3.3.3 Theme Three: [The Importance of] AYAs' Having a Voice in Palliative Care

##### 3.3.3.1 Subtheme 1: Gaps in Communication Interfere With AYAs' Decision‐Making

Thirteen primary studies, including 55 findings, revealed that AYA cancer survivors and their family members express a need for palliative care focused communication, which impacts quality of end‐of‐life care decision‐making [12, 33, 40, 45, 53, 54, 57, 72, 73, 74, 75, 76]. Currie et al. [54] for example, found “*the lowest scores indicating dissatisfactory care related to communication between the medical team and the patient/caregiver‐related to AYA illness and care outcomes*”. Similarly, another study identified that “*communication and decision‐making are a key end‐of‐life priority domain for [AYA cancer] patients' family members*” [72]. Consistently, suboptimal communication between AYA cancer patients, their caregivers, and the medical team has been demonstrated as a barrier to end‐of‐life communications. Specifically, for example, “*insensitivity to patients' needs, preferences, and values, [which leads to] insufficient discussions of prognosis and/or end‐of‐life, is a key communication‐related barrier.*” [53] Similarly, another study highlighted the significance of “*trusting staff and being* *supported by them” as a salient factor influencing patient's end‐of‐life care decision‐making.*” [57] Other factors have been evaluated and considered as factors impacting AYA cancer survivors and their family members' end‐of‐life decision‐making, such as faith, treatment options, and valuing quality‐of‐life, among others. These factors, however, were only reported in a couple of studies, falling short of emerging as a theme.

##### 3.3.3.2 Subtheme 2: Be Honest About End‐of‐Life Care Communication

Twelve primary studies, including 28 empirical findings, overwhelmingly recommended the medical team to have an *honest* conversation with AYA cancer patients and their family members about the patient's status of disease and prognosis [33, 35, 40, 48, 49, 52, 54, 57, 74, 75, 77, 78]. Studies have consistently reported that “*over three quarters (86%) of AYA cancer survivors prefer to know their prognosis*” [78], “*nearly half (45%) of caregivers wanted more information on what to expect at the time of death*” [54], and *“7% of parents and patients agreed on wanting to know if ‘I were dying’*” [35].

##### 3.3.3.3 Subtheme 3: Wanting to Be Involved and Heard in Palliative Care

Seven studies, inclusive of 16 empirical findings, revealed that AYAs, in general, want to be involved in their treatment choices and death options, with low patient‐parent congruence [35, 48, 57, 77, 79, 80, 81]. For example, studies have reported that *82% of teens [with cancer] prefer to complete an advance directive “that would let loved ones know my wishes if I were not able to speak for myself*” [35]. Despite feeling strongly about AYAs being involved in their end‐of‐life care decisions, limited findings revealed that parents and AYA cancer survivors may disagree on end‐of‐life‐related decisions. For example, one study found that “*family members and their adolescent [cancer patients] had poor congruence on ‘dying a natural death’*” [48].

#### 3.3.4 Theme Four: AYAs Choose Comfort and Peace for Palliative Care, Especially End‐of‐Life Care

##### 3.3.4.1 Subtheme 1: Managing a Plethora of Symptoms

Thirteen primary studies, including 29 primary findings, revealed a broad spectrum of symptoms commonly managed in palliative care for AYAs with cancer [32, 33, 34, 40, 49, 51, 56, 68, 70, 71, 72, 75, 82]. The most reported symptoms include pain management (13 empirical findings), mental health concerns (9 empirical findings), fatigue (4 empirical findings), and nausea and/or vomiting management (3 empirical findings).

##### 3.3.4.2 Subtheme: 2: The Value of Symptom Management Related to Comfort and Peace

Fifteen primary studies, including 33 primary findings, indicated that AYAs prefer their palliative care to prioritize life‐prolonging, physical comfort, pain palliation, and psychosocial support [12, 32, 35, 45, 48, 51, 53, 54, 69, 72, 75, 78, 80, 82, 83]. For example, one study identified that “*72% of patients or family surrogates preferred life‐prolonging care, … 20% wished for care focused on comfort, and 8% were undecided*” [32], and another study found that “*100% of teens [with cancer] prefer being at peace spiritually*” [35]. Another emerging theme reflected by four studies (inclusive of 4 empirical findings) suggested AYA's preference to be independent and have a sense of control in their cancer/palliative care [35, 48, 72, 75]. For example, one study reported that “*family members and their adolescents had excellent congruence on ‘saying everything I want to say to people in my family*’,” reflecting that both parents and patients want AYAs to be able to express themselves freely [48].

### 3.4 Other Notable Emerging/Trending Findings

#### 3.4.1 Trending Topic 1: AYA Palliative Care Interventions

Eleven studies, including 39 empirical findings, pertained to five different palliative care‐related interventions for AYA cancer [47, 69, 73, 75, 79, 81, 84, 85, 86, 87, 88]. The strongest evidence lies in the intervention of Family‐Centered Advance Care Planning for Teens with Cancer (FACE‐TC), with three primary studies and 12 empirical findings supporting its effectiveness in reducing depression and improving quality of life, among other outcomes [47, 79, 81]. Other interventions were each evaluated with limited evidence included the Voicing My CHOiCES Advance Care Planning Communication Guide [23], a Promoting Resilience in Stress Management tool (PRISM) [85, 87], an advance care planning video decision support tool [69], and a meaning‐centered program [84].

#### 3.4.2 Trending Topic 2: Limited Evidence on Positive Outcomes of Palliative Care in AYA Cancer

Despite an overwhelming level of evidence demonstrating the low quality and poor access to palliative care in AYA oncology, seven primary studies, including 13 primary findings, reported some, though limited, evidence on positive palliative care outcomes for AYAs with cancer [32, 36, 51, 54, 57, 68, 70]. One study found that “*special palliative care involvement was associated with improved pain trajectories (that are statistically significant)*” and “*most caregivers were satisfied with their AYA cancer decedent's end‐of‐life care;*” [70] though, again, these findings remain preliminary in terms of the number of supporting empirical evidence.

## 4 Discussion

Palliative care plays a critical role in supporting the short‐ and long‐term wellness of AYAs with cancer, but our understanding of palliative care for the AYA cancer population remains limited. This study, builds on the important work of Drake and colleagues [24], utilizing a meta‐thematic analytical framework to enhance our understanding of palliative care for AYAs with cancer. The study's findings extend what is already known about this topic and reveal important new insights regarding the latest state of the literature. A key finding of this study that has not been extensively evaluated by existing literature concerns the overall accessibility and quality of palliative care for AYAs with cancer. Existing evidence has described palliative care for the AYA cancer population and its benefits [21, 23] and suggested critical access barriers among the AYA cancer population. Findings of the current study concur with these important insights, with synthesized empirical evidence, suggesting not only access barriers but also the suboptimal quality of care among those receiving palliative care. Many empirical studies included in this review highlight the low percentage of AYA cancer patients who had access to palliative care, highlighting the numerous barriers to palliative care confronting the AYA cancer population, such as the institutional divide between pediatric and adult palliative care [25, 89] or AYA's perception of palliative care being equivalent to no cure [23, 90].

Consistent with the work by Drake and colleagues [24], existing evidence documenting the quality of palliative care mainly concerns end‐of‐life care, particularly discussion about death and documentation of do‐not‐resuscitate. Understandably, these are difficult conversations with patients of any age, but probably more so with AYAs, given this age cohort typically includes a sense of peak health and invincibility [91]. Findings of the current study offer important additional insights into AYA cancer patients' preference for having honest, though often difficult, conversations about their end‐of‐life care.

Another key finding of the study concerns the intensive measures and procedures delivered to AYAs with cancer, primarily during end‐of‐life care. End‐of‐life care is a core domain of palliative care intended to help patients live more comfortably during the last few weeks or months of life. Yet, existing empirical literature has consistently documented intensive procedures, high rates of hospitalization, and emergency department visits during many AYA cancer patients' last months of life [34]. Such a finding is consistent with what has been reported in the general palliative care literature, suggesting that aggressive care measures/procedures remain a salient gap and complex question in the palliative and end‐of‐life care field [92, 93].

A related key finding of this study is the identification of social drivers of health—specifically rurality, insurance status, low income, and racial and ethnic minority status—that contribute to the intensive measures of procedures in AYA palliative care. This finding aligns with a burgeoning body of literature highlighting the impact of social drivers on disparities in AYA palliative care delivery. AYAs from rural areas or with limited insurance coverage often face delayed referrals, restricted access to specialty palliative services, resulting in more aggressive end‐of‐life care [94]. Structural inequities—such as systemic racism and income‐related barriers—may further limit culturally responsive, developmentally appropriate care, particularly for minoritized populations [95]. These patterns underscore the need for targeted policy and practice reforms to ensure equitable, values‐congruent palliative care for all AYA patients, regardless of geographic or socioeconomic background.

Interestingly, the current study revealed that a diagnosis of hematological cancer has been linked with greater intensity of measures and care during end of life. This finding complements the work of Lockwood and colleagues, reporting cancer types and treatment protocols as important factors associated with different palliative care referral patterns [34]. Many hematological malignancies can be complex and often require palliative care early on. However, a previous study found that patients with hematological malignancies are more likely to encounter delayed timing of palliative care, resulting in services and treatments being more aggressive [96]. Future AYA oncology and palliative care focused research should attend to the use of aggressive measures during AYA cancer patients' last months of life and the delayed timing of palliative care in AYAs living with hematological malignancies.

Palliative care related communication is another important finding of the study, revealing a lack of palliative care focused communications that impact AYA cancer survivors' end‐of‐life decision‐making. Importantly, many AYA cancer patients and their family members expressed a preference to have thorough discussions with the care team, including prognosis, what to expect during end of life, and honest conversations about death. Yet, these communications are further complicated by a parallel finding that AYAs want to be involved in palliative care but have low congruency with their caregivers/parents in end‐of‐life care decision‐making. The unique age range of the AYA population has contributed to a greater involvement of caregivers/parents in their care decision‐making [97, 98]. The clinician‐parent‐patient relationship has long been established as a complicating factor for palliative care providers to deliver high‐quality care that advocates for AYA themselves while integrating voices from key stakeholders, such as their caregivers, for the best outcomes.

### 4.1 Limitations and Conclusion

Important limitations should be noted when considering study findings. First, there is always a possibility of missing eligible primary studies, and our search concluded in August 2024, leaving out eligible studies published since then. However, given the large number of studies and empirical findings included in the analysis, it is unlikely that a few missed articles would change any study findings. Second, most primary studies included in this review were not randomized controlled trials, limiting the level of evidence available for analysis. Future studies should consider meta‐analyzing palliative care interventions for the AYA cancer population using randomized controlled trial designs when enough studies become available. Finally, while meta‐thematic analysis is a notable strength of the current study, it is sensitive to researchers' inherent subjectivity, potentially leading to variations in findings. Despite our guard against this limitation by using multiple coders and comparing codes for objectivity and member affirmation, integrating perspectives from an AYA cancer survivor, the nature of meta‐thematic analytical findings should be noted.

Nevertheless, this study benefited from a comprehensive search strategy, structured meta‐thematic analysis to interpret empirical data, and a multidisciplinary team bringing different perspectives to data interpretation. The findings of the study offer an instrumental summary and update regarding the current state of empirical palliative care literature focusing on the AYA cancer population and identifying key areas of future research directions.

## Author Contributions

**Anao Zhang:** conceptualization, data curation, formal analysis, funding acquisition, methodology, project administration, resources, supervision, validation, visualization, writing – original draft, writing – review and editing. **Meiyan Chen:** data curation, formal analysis, methodology, writing – original Draft. **Rachel Brandon:** data curation, formal analysis, methodology, writing – original draft. **Jennifer Currin‐McCulloch:** data curation, methodology, writing – original draft, writing – review and editing. **Xingzhi Liu:** data curation, formal analysis, validation, writing – original draft. **Brennan Cheladyn:** methodology, validation, visualization, writing – review and editing. **Betty Ferrell:** formal analysis, supervision, writing – review and editing. **Barbara Jones:** formal analysis, supervision, writing – review and editing. **Bradley Zebrack:** formal analysis, supervision, writing – review and editing. **Adam S. DuVall:** formal analysis, supervision, writing – review and editing. All authors have contributed meaningfully to the paper that warrants authorship.

## Ethics Statement

The authors have nothing to report.

## Consent

The authors have nothing to report.

## Conflicts of Interest

The authors declare no conflicts of interest.

## Supporting information

Supporting Information S1

Supporting Information S2

## Data Availability

Data can be made available upon reasonable request to the corresponding author.
