# Supplementary material for: The Current State of Palliative Care Research for Adolescents and Young Adults With Cancer: A Systematic Review and Meta‐Thematic Analysis of Empirical Literature
Source: Psychooncology. 2025 Jul 14;34(7):e70228. doi: 10.1002/pon.70228 (PMC12260280; doi:10.1002/pon.70228)
Supplement: Supplementary file 1 — Supporting Information S1 [file PON-34-e70228-s002.docx]

**Supplement 1. Search Strategies and Details**

Search Strategy

**Databases**

We included the following databases: Academic Search Complete, APA PsycInfo, AMED, CINAHL Complete, Global Health, Health Policy Reference Center, APA PsycArticles, Social Sciences Abstracts, Medline, and Cochrane Library.

**Interfaces**

We used the Ovid platform for Medline and the Cochrane website for the Cochrane Library database. Other than the two above-mentioned databases, we used the EBSCO platform to conduct cross-database searches for the remaining databases indicated earlier.

**Grey literature**

We searched for professional websites, including the American Psychosocial Oncology Society website, the Association of Oncology Social Work website, the American Academy of Hospice and Palliative Medicine website, and the European Palliative Care Academy website.

**Inclusion and exclusion criteria**

**Inclusion criteria:**

• Only empirical studies are eligible for inclusion.

• Published in English language.

• Focus on AYAs with cancer – OK to include pediatrics but must include adolescent and/or young adult

• A study should have direct connection with palliative care in AYAs with cancer, e.g., understanding the differential experiences of AYAs versus non-AYAs receiving palliative care, evaluating factors impacting the uptake of palliative care, testing different approaches of delivering palliative care to AYAs with cancer, or qualitatively evaluating oncologists’ perspectives on palliative care delivery to AYAs with cancer.

• Common study designs include cross-sectional or longitudinal empirical study, controlled trial studies (either true experimental or quasi-experimental), or a chart review.

Exclusion criteria:

• Non-empirical but purely theoretical or conceptual

• Studies primarily report the benefit of palliative care for AYAs with cancer or the consequences of not delivering palliative care to AYAs with cancer.

• Studies primarily report AYA cancer survivors’ experiences of receiving palliative care without direct connection to care/service delivery, such as a case study, will be excluded unless the study has direct connection with palliative care delivery for AYA cancer.

• General focus on life-limiting conditions

• Just advance cancer AYA population without reference to end-of-life or palliative care

**Query strings**

**• Search keywords (for non-medical database):**

**AYA-related keywords:** “adolescen*” or “young adult” or “young” or “teen*” or “youth” or “AYA”

**Palliative-related keywords:** “palliative” or “end-of-life” or “end of life” or “eol” or “hospice”

**Cancer-related keywords:** “cancer” or “oncolog*” or “neoplasms” or “hematolog*”

**• Search keywords (MeSH Term)**

**AYA-related keywords:** “adolescent” or “adolescence” or “teen” or “teenager” or “youth”

**Palliative-related keywords:** “palliative” or “hospice”

**Cancer-related keywords:** “tumor” or “neoplasm” or “cancer” or “malignancy” or “malignancies”

**Search validation procedure**

In addition to a comprehensive and systematic search strategy, we will also have known experts in the field of palliative care and AYA cancer to review the list of included studies. In addition, we also scanned existing review papers, theoretical, conceptual, or empirical, on the AYA palliative care to confirm if we missed any important studies.

**Other search strategies**

As indicated earlier, we also screened for reference lists in published review papers focusing on palliative care and AYA oncology.

**Procedures to contact authors**

Given the nature of our analysis, it is not applicable for us to contact the authors.

**Results of contacting authors**

Not Applicable.

**Search expiration and repetition**

We follow the Cochrane guideline and will update the search after 12 months since the initial search date.

**Search strategy justification**

Our search of the keywords contains three broad areas to identify the three relevant substantive topics, i.e., adolescent and young adult (age), cancer (diagnosis), and palliative care (intervention). We consider this the most balanced approach because it allows us to have the broadest, i.e., most inclusive approach, to search for the most relevant studies. Our complementary approach includes grey literature and possible references on professional websites, which would ensure that our focused approach is the most comprehensive and inclusive of possible references.

**Miscellaneous search strategy details**

Not applicable
