# Supplementary material for: The Current State of Palliative Care Research for Adolescents and Young Adults With Cancer: A Systematic Review and Meta‐Thematic Analysis of Empirical Literature
Source: Psychooncology. 2025 Jul 14;34(7):e70228. doi: 10.1002/pon.70228 (PMC12260280; doi:10.1002/pon.70228)
Supplement: Supplementary file 2 — Supporting Information S2 [file PON-34-e70228-s001.docx]

| **Supplemental Study Table** | | | | | | | | | | |
| --- | --- | --- | --- | --- | --- | --- | --- | --- | --- | --- |
| **Author** | **Pub Year** | **Study Type** | **Data Set** | **Country** | **Participant Type** | **Age Range** | **Mean Age** | **Palliative Care Definition** | **Assessment**  **Tool*** | **Overall RoB** |
| Abdelaal et al. | 2021 | Chart Review | Medicaid - Integrated AYA Palliative Care and Psychiatry Clinic (IAPCPC) | Canada | AYA = Yes  Caregiver = No  Provider = No | 17-39 | 31.52 |  | QATOCCS | Low |
| Ananth et al. | 2021 | Interview | qualitative interviews and focus groups | United States | AYA = Yes  Caregiver = Yes  Provider = Yes | 16-25 | 20 |  | QATOCCS | Moderate |
| Arruda-Colli et al. | 2018 | Survey | Primary data collection | Australia and Brazil | AYA = Yes  Caregiver = Yes  Provider = Yes | 15-39 |  |  | QATOCCS | High |
| Avery et al. | 2020 | Text analysis | Primary data collection | Canada | AYA = Yes  Caregiver = No  Provider = No | 21-39 | 31.25 | Palliative care is defined as care that improves the  quality of life of patients and their caregivers at any  stage of a life-threatening illness. | QATOCCS | Low |
| Baker et al. | 2022 | Intervention | Primary data collection | United States | AYA = Yes  Caregiver = Yes  Provider = No | 14-20 | 16.9 |  | QACIS | Low |
| Bell et al. | 2010 | Chart Review | Medicaid | United States | AYA = Yes  Caregiver = No  Provider = No | 10-21 | 14.4 |  | QATOCCS | Moderate |
| Cicero-Oneto | 2017 | Survey | Primary data collection | Mexico | AYA = Yes  Caregiver = Yes  Provider = Yes | 13-18 |  | ...are those medical interventions that do not attempt to cure, but rather try to alleviate the discomfort, pain, and suffering. | QACSS | Moderate |
| Cohen-Gogo et al. | 2011 | Chart Review | Primary data collection | United States | AYA = Yes  Caregiver = No  Provider = No | 5.2-24.2 | 15.9 |  | QACSS | Low |
| Coltin et al. | 2022 | Chart Review | Initiative to Maximize Progress in Adolescent and Young Adult Cancer Therapy (IMPACT) | Canada | AYA = Yes  Caregiver = No  Provider = No | 18-21 | 20 |  | QATOCCS | Low |
| Currie et al. | 2022 | Survey | Primary data collection | United States | AYA = No  Caregiver = Yes  Provider = No |  |  |  | QATOCCS | Low |
| Devlin et al. | 2019 | Chart Review | EMR | United States | AYA = Yes  Caregiver = No  Provider = No | 18-39 |  |  | QATOCCS | Moderate |
| Fernando et al. | 2024 | Quality Improvement |  | United Kingdom | AYA = Yes  Caregiver = No  Provider = No | 15-39 |  |  | QABAS | Moderate |
| Fladeboe et al. | 2021 | Intervention | Primary data collection | United States | AYA = Yes  Caregiver = No  Provider = No | 12-22 | 16 |  | QATOCCS | Low |
| Fletcher et al. | 2018 | Chart Review | EMR | Australia | AYA = Yes  Caregiver = No  Provider = No | 15-25 | 19.9 |  | QACCS | Low |
| Foster et al. | 2019 | Chart Review | Medicaid | United States | AYA = Yes  Caregiver = No  Provider = No | 18-39 | 30.8 | The goal of palliative care (PC) is to anticipate, prevent, and reduce suffering associated with an advanced disease while supporting the best possible quality of life for patients and their families regardless of the need for other therapies. In oncology, the integration of early PC with standard care has been shown to improve quality of life, improve patient satisfaction, decrease aggressive care at the end of life (EOL), increase communication about EOL care wishes, and improve overall survival. | QATOCCS | Low |
| Friebert et al. | 2020 | Survey | Primary data collection | United States | AYA = Yes  Caregiver = Yes  Provider = No | 14-20 | 16.9 |  | QATOCCS | Moderate |
| Grinyer et al. | 2004 | Text analysis | Secondary data analysis of narrative material | United Kingdom; Germany and Australia | AYA = No  Caregiver = Yes  Provider = No |  |  |  | QACSS | High |
| Gupta et al. | 2023 | Chart Review | Medicaid | Canada | AYA = Yes  Caregiver = No  Provider = No | 22-27 |  |  | QATOCCS | Low |
| Hasegawa et al. | 2023 | Chart Review | Medicaid | Japan | AYA = Yes  Caregiver = No  Provider = No | 15-39 |  |  | QATOCCS | Moderate |
| Hinds et al. | 2005 | Survey | Primary data collection | Australia | AYA = Yes  Caregiver = Yes  Provider = Yes | 10-20 | 17.4 |  | QACSS | Low |
| Hirano et al. | 2019 | Survey | Primary data collection | Japan | AYA = Yes  Caregiver = No  Provider = No | 15-39 |  |  | QATOCCS | Moderate |
| Hughes et al. | 2015 | Chart Review | Medicaid | Australia | AYA = Yes  Caregiver = No  Provider = No | 16-24 | 21.5 |  | QACCS | Low |
| Jacobs et al. | 2015 | Survey | Primary data collection | United States | AYA = Yes  Caregiver = Yes  Provider = Yes | 14-21 | 16 |  |  | Low |
| Jewitt et al. | 2023 | Survey | Secondary data analysis | Canada | AYA = Yes  Caregiver = No  Provider = No | 15-39 |  |  | QATOCCS | Low |
| Johnston et al. | 2017 | Chart Review | OSHPD | United States | AYA = Yes  Caregiver = No  Provider = No | 15-39 |  |  | QATOCCS | Low |
| Johnston et al. | 2018 | Chart Review | OSHPD | United States | AYA = Yes  Caregiver = No  Provider = No | 15-39 |  |  | QATOCCS | Low |
| Kang et al. | 2015 | Survey | Primary data collection | South Korea | AYA = Yes  Caregiver = No  Provider = No |  | 24.9 |  | QABAS | Moderate |
| Kassam et al. | 2021 | Chart Review | Ontario Cancer  Registry | Canada | AYA = Yes  Caregiver = No  Provider = No | 15-44 |  |  | QATOCCS | Low |
| Katz et al. | 2022 | Chart Review | EMR | Australia | AYA = Yes  Caregiver = No  Provider = No | 15-27 |  |  | QACSS | Low |
| Keim-Malpass et al. | 2014 | Chart Review | Medicaid | United States | AYA = Yes  Caregiver = No  Provider = No | 17-39 | 30.7 |  | QATOCCS | Low |
| Lau et al. | 2020 | Intervention | Primary data collection | United States | AYA = Yes  Caregiver = No  Provider = No | 12-25 |  |  | QACIS | Low |
| Lockwood et al., | 2021 | Chart Review | Medicaid | United States | AYA = Yes  Caregiver = No  Provider = No | 18-39 |  | Palliative care (PC) serves a valuable role to address these unmet needs with its specialized care for patients living with serious illness, supporting a desired quality of life and alleviating physical, social, psychological, and spiritual distress irrespective of disease status. | QACSS | Low |
| Lyon et al. | 2013 | Intervention | Primary data collection | United States | AYA = Yes  Caregiver = Yes  Provider = No | 14-21 | 16.3 |  | QACIS | Moderate |
| Lyon et al. | 2014 | Intervention | Primary data collection | United States | AYA = Yes  Caregiver = Yes  Provider = No | 14-21 | 16.3 | Palliative care (PC) currently exists in the context of a changing health care delivery system in which death is medicalized. | QACIS | Low |
| Mack et al. | 2015 | Chart Review | New York State Cancer Registry and state Medicaid program | United States | AYA = Yes  Caregiver = No  Provider = No | 15-29 |  |  | QATOCCS | Low |
| Mack et al. | 2015 | Chart Review | Kaiser Permanente Southern California (KSPC) cancer registry data and electronic health records | United States | AYA = Yes  Caregiver = No  Provider = No | 15-39 |  |  | QATOCCS | Low |
| Mack et al. | 2016 | Chart Review | EMR | United States | AYA = Yes  Caregiver = No  Provider = No | 15-39 |  |  | QATOCCS | Low |
| Mack et al. | 2021 | Survey | Primary data collection | United States | AYA = Yes  Caregiver = Yes  Provider = Yes | 12-39 |  |  | QACSS | Low |
| Mack et al. | 2021 | Text analysis | Primary data collection | United States | AYA = Yes  Caregiver = Yes  Provider = No |  |  | Palliative care as an approach to care focused on improving quality of life for patients with life-threatening illness, and defined hospice as a type of healthcare, often provided in the home but sometimes  also available as an inpatient experience, that is focused on treating pain, symptoms, and emotional and spiritual distress for those nearing EoL. | QACSS | Low |
| Mark et al. | 2019 | Chart Review | Medicaid | United States | AYA = Yes  Caregiver = No  Provider = No | 20-27 | 24.2 |  | QATOCCS | Low |
| Montel et al. | 2009 | Survey | Primary data collection | France | AYA = No  Caregiver = Yes  Provider = No | aya(15-25) |  |  | QACSS | Low |
| Mooney-Doyle et al. | 2022 | Survey | Medicaid | United States | AYA = Yes  Caregiver = No  Provider = No | 20-20 | 20 |  | QACCS | Low |
| Mori et al. | 2018 | Survey | Primary data collection | Japan | AYA =  Caregiver = Yes  Provider = | 20-39 | 34 |  | QATOCCS | Moderate |
| Mun et al. | 2021 | Chart Review | Medicaid | United States | AYA = Yes  Caregiver = No  Provider = No | 0-39 |  |  | QATOCCS | Low |
| Murray et al. | 2021 | Chart Review | EMR | United Kingdom | AYA = Yes  Caregiver = No  Provider = No | 16-25 |  |  | QACSS | Low |
| Needle et al. | 2022 | Intervention | Medicaid | United States | AYA = Yes  Caregiver = Yes  Provider = No | 14-20 | T: 16.9  C: 17.0 |  | QACIS | Low |
| Noh et al. | 2023 | Chart Review | Medicaid | United States | AYA = Yes  Caregiver = No  Provider = No | 15-39 |  |  | QATOCCS | Low |
| Poort et al. | 2020 | Chart Review | OSHPD | United States | AYA = Yes  Caregiver = No  Provider = No | 18-34 | 28.09 |  | QACSS | Low |
| Pousset et al. | 2009 | Survey | Primary data collection | Belgium | AYA = Yes  Caregiver = No  Provider = No | 11-18 | 14.8 |  | QATOCCS | Moderate |
| Rajeshuni et al. | 2017 | Chart Review | Primary data collection | United States | AYA = Yes  Caregiver = No  Provider = No | 15-39 |  | Palliative care is a holistic approach that improves the quality of life of patients and family members facing life-threatening illness. | QATOCCS | Low |
| Revon‐Rivière et al. | 2019 | Chart Review | Medicaid | France | AYA = Yes  Caregiver = No  Provider = No | 0-25 |  |  | QATOCCS | Low |
| Roeland et al. | 2020 | Chart Review | Medicaid | United States | AYA = Yes  Caregiver = No  Provider = No | 15-39 |  | Palliative care offers expertise and resources for symptom control and quality of life for patients with cancer regardless of prognosis. | QATOCCS | Low |
| Sansom-Daly et al. | 2023 | Intervention | Primary data collection | Australia | AYA = Yes  Caregiver = Yes  Provider = Yes | 16-21 | 19.33 |  | QATOCCS | Low |
| Sisk et al. | 2022 | Survey | Secondary data analysis | United States | AYA = No  Caregiver = Yes  Provider = No | 15-39 |  |  |  | Low |
| Snaman et al. | 2017 | Chart Review | Medicaid | United States | AYA = Yes  Caregiver = No  Provider = No | 14.8-25.8 | 18.4 | The primary tenets of palliative care (PC) include providing high-quality communication, advance care planning, and assessing and treating physical and psychological symptoms in the context of an interdisciplinary team, with the goal of improving support for, and reducing the suffering of patients and caregivers. | QATOCCS | Low |
| Snaman et al. | 2023 | Intervention | Primary data collection | United States | AYA = Yes  Caregiver = Yes  Provider = No | 18-39 | 33 | ...the 3-goal framework commonly used in pediatric palliative care: life-prolonging care (ie, live as long as possible), selective care (ie, live as long and as well as possible), and comfort care (ie, live as comfortably as possible). | QACIS | Low |
| *** RoB = Risk of Bias**  *** Assessment Tool：**  QACIS = Quality Assessment of Controlled Intervention Studies  QATOCCS = Quality Assessment Tool for Observational Cohort and Cross-Sectional Studies  QACCS = Quality Assessment of Case-Control Studies  QABAS = Quality Assessment Tool for Before-After (Pre-Post) Studies With No Control Group  QACSS = Quality Assessment Tool for Case Series Studies | | | | | | | | | | |
